# Postprandial Bioactivity of a Spread Cheese Enriched with Mountain Tea and Orange Peel Extract in Plasma Oxidative Stress Status, Serum Lipids and Glucose Levels: An Interventional Study in Healthy Adults

**DOI:** 10.3390/biom11081241

**Published:** 2021-08-19

**Authors:** Olga Papagianni, Konstantina Argyri, Thomas Loukas, Athanasios Magkoutis, Theodora Biagki, Dimitrios Skalkos, Dimitrios Kafetzopoulos, Charalampia Dimou, Haralampos C. Karantonis, Antonios E. Koutelidakis

**Affiliations:** 1 Laboratory of Nutrition and Public Health, Human Nutrition Unit, Department of Food Science and Nutrition, University of the Aegean, 81400 Myrina, Lemnos, Greece; olga3_pap@yahoo.gr (O.P.); nargiri@gmail.com (K.A.); theodorabiagki@gmail.com (T.B.); chadim@aegean.gr (C.D.); 2 Outpatιent Clinic, 81400 Myrina, Lemnos, Greece; tloukas2002@yahoo.com (T.L.); tmagoutis@gmail.com (A.M.); 3 Laboratory of Food Chemistry, Department of Chemistry, University of Ioannina, 45110 Ioannina, Greece; dskalkos@uoi.gr; 4 Department of Business Administration, University of Macedonia, 54636 Thessaloniki, Greece; dimkafe@uom.edu.gr; 5 Laboratory of Food Chemistry, Biochemistry and Technology, Department of Food Science and Nutrition, University of the Aegean, 81400 Myrina, Lemnos, Greece; chkarantonis@aegean.gr

**Keywords:** postprandial bioactivity, bioactive-compounds-enhanced cheese, orange peel extract, mountain tea extract, metabolic biomarkers

## Abstract

Postprandial lipemia, glycemia and oxidative stress may affect the occurrence of cardiovascular disease. The purpose of the present intervention study was to investigate the effect of a spread cheese enriched with mountain tea (*Sideritis* sp.) and orange peel (*Citrus sinensis*) extract on postprandial metabolic biomarkers in healthy volunteers. In a cross-over design, 14 healthy subjects 20–30 years old were consumed either a meal rich in fat and carbohydrates (80 g white bread, 40 g butter and 30 g full fat spread cheese) or a meal with the spread cheese enriched with 6% mountain tea–orange peel extract. Differences in postprandial total plasma antioxidant capacity, resistance of plasma to oxidation, serum lipids, glucose and uric acid levels were evaluated at 0, 1.5 and 3 h after consumption. Plasma total antioxidant capacity was significantly increased 3 h after the consumption of the meal in the presence of the extract-enriched cheese, compared to the conventional cheese (*p* = 0.05). Plasma resistance to oxidation was increased at 30 min in the Functional meal compared with the Control meal. A tendency to decrease the postprandial rise in glucose and triglyceride levels, 1.5 h and 3 h, respectively, after the intake of the meal with the extract-enriched cheese was observed (*p* = 0.062). No significant changes in the concentrations of the remaining biomarkers studied were observed (*p >* 0.05). Further studies with a larger sample are needed in both healthy adults and patients with cardiovascular disease to draw safer conclusions about the postprandial effect of the extracts on metabolic biomarkers that predict cardiovascular risk.

## 1. Introduction

Chronic diseases, including cardiovascular disease and diabetes, can lead to hospitalization, long-term disability and reduced quality of life, while persistent conditions are ranked among the top causes of global deaths. Postprandial inflammation and oxidative stress may have a significant effect on the risk of these diseases [1]. The postprandial state occurs after meal ingestion and incorporates the digestion and absorption of nutrients and biomolecules (6–12 h). In this complex and dynamic state, almost all tissues and organs are involved since the human body, apart from nutrients, is also exposed to factors that influence metabolism, inflammation and overal health. The ingestion of various dietary components influences postprandial inflammation via different pathways [2]. Although the underlying mechanisms are not fully elucidated, the consumption of a meal rich in fat or carbohydrates or their combination triggers the appearance of dysfunctions that include oxidative stress, low-grade inflammation and endothelial dysfunction, all relating to the acute rise of postprandial plasma lipids and glucose [3].

Under normal circumstances, postprandially, there is a rapid increase in plasma glucose concentration, as the rate of glucose absorption is higher than the rate of endogenous glucose production. The amount of food consumed and its composition, the consumption duration, the carbohydrate content, the rate of glucose absorption and insulin resistance are important factors that affect the degree of postprandial glycemia. The concentration of glucose in plasma, 2 h after consuming a meal, is a key factor in predicting the risk of cardiovascular disease [4]. Postprandial lipemia, on the other hand, is affected by changes in plasma lipids composition and concentration. Hyperlipemia is attributed to the blood accumulation of triglyceride-rich lipoproteins (TRL), synthesized by the liver’s very-low-density lipoproteins (VLDL) and intestinal chylomicrons (CM) [5]. Factors contributing to this accumulation are the overproduction of both VLDL and CM and a defective TRL removal process. Endogenous factors, including circulating hormones or free fatty acids, genetic variants and exogenous factors including food components and food structure, may affect postprandial lipemia in humans [6].

Excessive increase in plasma glucose and lipids inhibits post-meal oxidative phosphorylation of mitochondria, causing increased simple electrons’ transfer to molecular oxygen. Thus, increasing levels of superoxide anions enter the circulation. Furthermore, under these conditions, the production of Reactive Oxygen Species (ROS) by leukocytes is promoted. The key factor influencing the degree of postprandial oxidative stress is the amount of caloric intake [7]. In addition, elevated postprandial glucose and lipid levels induce the activation of various inflammatory pathways through the increase in the production of cytokines and other pro-inflammatory mediators [2,8]. Scientific evidence suggests that low-grade inflammation and endothelial dysfunction, in combination with insulin resistance, have been associated with increased cardiovascular risk, including metabolic syndrome (MS), cardiovascular disease (CVD) and type 2 diabetes [3]. Taken together, the postprandial state following a meal offers an interesting tool to study the impact of nutrients on adverse postprandial effects and predict the development of possible cardiometabolic risk factors.

Various epidemiological and clinical studies in recent years indicate that people who adopt a diet rich in fruits, vegetables, raw cereals, fish and dairy products low in saturated fat have a reduced cardiovascular risk. However, such a diet requires significant changes in eating habits and consumer behavior and frequently does not fit to typical Western diet [9]. Recent scientific evidence suggests a possible beneficial effect of functional foods consumption on reducing the risk and progress of chronic diseases, such as obesity, cardiovascular disease and diabetes [10]. Functional foods are defined as natural or processed foods, which have been proven, based on scientific studies, that due to their bioactive constituents contribute to the achievement of specific functional targets within the body, leading to possible disease prevention and health promotion [11,12]. Innovative functional foods are enriched with bioactive compounds, which have been suggested for their contribution to reducing the risk of cardiovascular disease, metabolic syndrome and type-2 diabetes. Among bioactive compounds studied, antioxidants as polyphenols, phytosterols and phytostanols, carotenoids, dietary fiber, e.g., β-glucans, oligosaccharides, e.g., fructans and n-3 long-chain fatty acids, have been shown to play an important role both in decreasing postprandial lipid levels and reducing glycemic response and oxidative stress [9,10]. Subsequently, several dietary interventions, which include functional meals, have been suggested as possible contributors to diseases’ risk factor’s control [13].

In the present study, we tested the hypothesis that the addition of bioactive compounds, mainly polyphenols, to a challenge meal (high in fat and carbohydrates), induces favorable effects in the postprandial state. Polyphenols may exhibit antioxidative and anti-inflammation effects through strong radical-scavenging properties, inhibit the development of hyperlipidemia [14], improve glycemic control [15] and exert activity against glycation and glycoxidation [16]. Bioactive compounds derived from natural functional foods and food process by-products were used as candidates for acute protective effects during the postprandial phase since limited scientific data are available on potential postprandial activity of innovative foods enriched with such bioactive compounds. It has been reported that frequent consumption of mountain tea (*Sideritis* sp.) contributes to insulin resistance prevention by lowering both plasma glucose and lipid levels. In addition, mountain tea has shown antioxidant potential due to its non-enzymatic, natural content of antioxidants, as well as its ability to increase the action of catalase into the liver [17,18]. In addition, orange peel flavonoids have been reported as natural antioxidants and anti-inflammatory promoters, but knowledge on the accurate action mechanism is limited [19]. The incorporation of flavonoids from orange peels constitutes a strategy for the valorization of these industrial by-products that satisfies sustainable development principles [20]. Findings from clinical trials-dietary interventions show that the consumption of a meal containing a spread cheese may cause a significant increase in the triglyceride response postprandially, 2 h after the consumption of the meal [21]. In vitro data show that polyphenol-enriched cheese protects polyphenol integrity and antioxidant activity in the gastrointestinal environment [22]. To our knowledge, this is the first study that investigates the acute effects of the consumption of a cheese enriched with bioactive compounds derived from natural functional food and by-product sources on the postprandial state in healthy subjects.

The objective of this study was to investigate, in healthy adults, the acute postprandial bioactivity after consumption of a spread cheese enriched with mountain tea *(Sideritis* sp.) and orange peel extract on lipidemic and oxidative biomarkers, which are correlated with chronic diseases such as cardiovascular disease and metabolic syndrome.

## 2. Materials and Methods

### 2.1. Subjects

The study was carried out at Human Nutrition Unit of the Department of Food Science and Nutrition of the University of the Aegean. The study protocol was reviewed and approved by the University of the Aegean Ethics Committee (No. 7505, 20 October 2019). It was conducted in accordance with the ethical standards laid down in the Declaration of Helsinki and followed the Principles of Good Clinical Practice. The staff of the study provided detailed information regarding the aims, methods, anticipated benefits of the study, the confidentiality of the data and the voluntary nature of participation. Written consent was obtained from each subject before participation.

A total of 14 (*n* = 14) healthy volunteers, 6 men and 8 women, aged 20–30 years old, from Lemnos Island, Greece, were recruited from February 2020 to January 2021 to participate in this study, after an initial screening of 18. All volunteers were initially screened by completing a questionnaire recording medical history, demographic characteristics, frequency of consumption of foods rich in polyphenols, level of physical activity and general habits, as smoking and alcohol consumption 6 months prior to the study. Anthropometric data were also collected. Exclusion criteria were age over 30 years old, dietary supplements consumption during the last 2 months, history of chronic illness including type I and II diabetes (hemoglobin A1c—HbA1c > 5%), moderate or heavy smokers (>10 cigarettes/day), abnormal Body Mass Index (BMI) (>25 kg/m^2^) and alcohol overconsumption (>40 g alcohol/day). The volunteers were also screened by biochemical blood tests, in collaboration with extramural doctors, to exclude cases with abnormal hematological and biochemical profiles (cholesterol > 6.8 mM, triglycerides > 2.8 mM, glucose > 6.11 mM). Subjects were advised to refrain from taking medication or dietary supplements on the days of the study and abstain from consuming foods high in antioxidants and alcohol for 24 h before the study.

### 2.2. Characterization of the Enriched Spread Cheese Preparation

The spread cheese used in this study was a commercial full-fat product (donated from AMFIGAL S.A., Amfilochia, Greece). The choice of the extract ingredients was based on in vitro comparison of antioxidant capacity and polyphenol content among 20 combinations of Greek herbs and fruit by-products. In addition, sensorial evaluation was conducted in a small sample of 10 volunteers in six produced kinds of cheese with added combinations: thyme–orange peel, origanum–orange peel, mountain tea–olive leaves, mountain tea–origanum, thyme–olive leaves, origanum–olive leaves, as well as measurement of the antioxidant properties of the tested cheeses. The results led to the selected combination of mountain tea with orange peel due to both increased antioxidant capacity and improved, accepted organoleptic characteristics (data do not show)**.** Therefore, the infusion used for the enrichment consisted of mountain tea (*Sideritis sp.*) and orange peel and was prepared by ultrasonic extraction (70 °C, 1 h) by adding 2 g of herb and 10 g of orange peel in 100 mL distilled water. The novel cheeses were prepared by mixing 1.2 mL of the infusion with 6.8 g of spread cheese. The final concentration was 6% mountain tea–orange peel extract into the enriched cheese.

Total antioxidant capacity of the infusions and the novel spread cheeses was measured using the Ferric Reducing Antioxidant Power (FRAP) method performed in 96-well flat-bottom microplates (Merck KGaA, Darmstadt, Germany) [23]. In brief, FRAP reagent containing 300 mM acetate buffer pH 3.6, 10 mM 2,4,6-tri(2-pyridyl)-*S*-triazine solution in 40 mM HCl and 20 mM FeCl_3_·6H_2_O solution in a ratio of 10:1:1 was incubated at 37 °C before use. A total of 20 μL of sample was mixed with 80 μL FRAP reagent and incubated at room temperature (25 °C) for 30 min in a dark environment. The reaction was measured by monitoring in a plate reader (Spark^®^ Multimode Microplate Reader, Tecan, Merck KGaA, Darmstadt, Germany) at a wavelength of 595 nm. Standards were prepared from FeSO_4_ in HCl 0.01 N in concentrations ranging from 100 to 1000 mM. The antioxidant capacity was expressed quantitatively as micromoles FeSO_4_/milliliter (μmol/mL), based on FeSO_4_·7H_2_O calibration curve.

Total phenolic content of the infusions and the novel cheeses was evaluated according to the Folin–Ciocalteu assay [24]. Folin–Ciocalteu assay was performed by mixing 20 μL of 7.5% (*v*/*v*) Na_2_CO_3_, 50 μL of each sample and 20 μL of Folin–Ciocalteu reagent in 96 well- flat-bottom microplates. The samples were incubated for 30 min at room temperature in the dark and the spectrophotometric determination was performed at 765 nm in the plate reader. The total phenolic content of samples was expressed as micrograms of gallic acid equivalents (GA) per milliliter (μg GA/mL) of liquid matter, based on a gallic acid calibration curve.

All chemicals were purchased from Sigma-Aldrich (Steinheim, Germany). Double distilled, deionized water was used throughout the experiments.

### 2.3. Meals

The Control meal consisted of two slices of white bread (procured by a local bakery), 80 g (2 slices of 40 g), two bars of unsalted butter (Lurpak, Arla Foods, Aarhus, Denmark), 20 g (2 bars of 10 g) and spread cheese, 30 g (2 individual portions of 15 g). The composition of the Functional meal was the same as the Control, except that it contained the enriched spread cheese. Each whole meal totally weighed 130 g. Dietary composition of the Control meal is shown in Table 1.

### 2.4. Study Design

This was a two-period, cross-over, acute interventional study. Subjects were scheduled at a minimum of 1 week between treatments to ensure an adequate washout period for the next intervention. On enrolment, all subjects were randomly assigned to the C: F group (Control: Functional) or to the F: C group (Functional: Control). Subjects in the C: F group received the Control meal on the first visit and the Functional meal on the second visit, while those in the F: C group received the Functional meal on the first visit and Control meal on the second visit.

**Figure 1:**
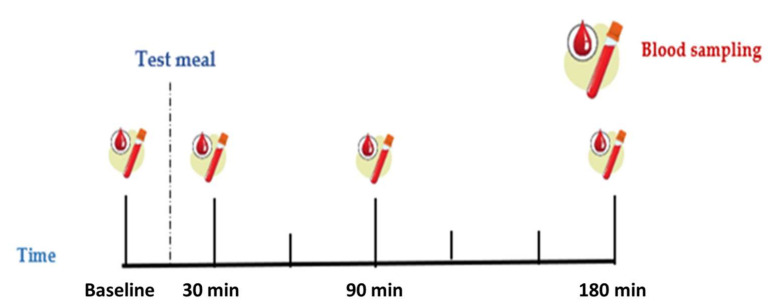
Test visit flow diagram.

Participants were admitted to the Human Nutrition Unit on two separate occasions (Control meal, Functional meal) at 09:00 a.m., after a 12 h overnight fast. Upon their arrival, they were asked to fill out a short recall questionnaire referring to the last 24 h and then fasting blood sample (0 min—baseline) was collected from forearm vein with intravenous catheter by a doctor. Subsequently, subjects were given 130 g of the Control or the Functional meal, depending on their group, and were asked to consume it within 15 min. Blood samples (10 mL) were drawn at 30, 90 and 180 min following the ingestion of the meal (Figure 1). During the trial, one cup of water (250 mL) was available for each subject.

Blood samples were taken into Ethylenediaminetetraacetic acid (EDTA) and citric acid-treated tubes for plasma separation or heparinized tubes for serum separation. Plasma or serum was separated by centrifugation for 10 min at 3500× *g* in a clinical centrifuge immediately after blood collection. Aliquots of plasma or serum were stored at −40 °C until analysis.

### 2.5. Chemical Analysis of Blood Samples

#### 2.5.1. Total Antioxidant Capacity and Resistance of Plasma to Copper-Induced Oxidation

Plasma total antioxidant capacity was measured using the FRAP method as described above. The resistance of plasma to copper-induced oxidation was performed as previously described by Karantonis et al. [25]. Briefly, 15 μL of plasma was added to 660 μL phosphate-buffered solution, pH 7.4, 146 mM in NaCl, and the oxidation reaction was started by the addition of 75 μL CuSO_4_ 1 mM. The absorbance of the resulting conjugated dienes that lead to sigmoid curves was recorded at 245 nm for 3 h at 37 °C on a Spectrophotometer Lambda 25 (Perkin-Elmer, Norwalk, CT, USA). Resistance of plasma oxidation is evaluated by the value of lag time (LTPOX, Lag Time of Plasma Oxidation), which is calculated from the time corresponding to the intersection of the tangents of the initial and acceleration periods of the sigmoid curves.

#### 2.5.2. Biochemical Parameters

Blood glucose, total cholesterol, high-density lipoprotein cholesterol (HDL), low-density lipoprotein cholesterol (LDL), triglycerides and uric acid were measured in serum with an automated analyzer (COBAS c111, Roche, Switzerland).

### 2.6. Statistical Analysis

Statistical analysis was performed by SPSS (SPSS V21.0) and Prism 9 (GraphPad Software Inc., San Diego, CA, USA). Sample calculation power was calculated for the primary outcome and venous plasma antioxidant capacity (TAC) using Statmate version 2.0 (GraphPad Software Inc.). Taking an α = 0.01, a sample size of 14 subjects allows for detection of a difference of 0.21 mmol TAC/L between groups, calculated from an expected SD of between-meal group differences of 0.21 mmol/L. Results are presented as means ± SD, and significance was at *p* < or = 0.05. Before any statistical analysis, all variables were tested for normal distribution. Repeated measures ANOVA (Analysis of Variance) and Bonferroni post-tests were performed on differences between plasma and serum samples at 30 min, 1.5 and 3 h, for each meal group and on change from baseline data for venous plasma TAC and serum biomarkers. Differences between the two treatment groups at any time and time period from baseline were also tested by paired *t*-tests. An incremental Area Under Curve (iAUC) was calculated using the trapezoidal method and ignoring the area below baseline. Differences between iAUCs for each treatment were tested by Repeated ANOVA. The variables followed a normal distribution.

## 3. Results

Preliminary results of the pilot study have been presented at the First International Electronic Conference on Food Science and Functional Foods (Foods 2020) and shortly published on the conference proceedings [26].

### 3.1. In Vitro Antioxidant Capacity and Total Phenolic Content of Spread Cheeses and Infusion Extract

According to the in vitro experiments, the mountain tea–orange peel extract provided a total antioxidant capacity of 34.67 μmol FeSO_4_/mL, while the novel cheese provided 1.22 μmol FeSO_4_/g. Furthermore, the total phenolics of the extract were determined as 49.9 μg GA/mL, while the total phenolics of the novel cheese were 6.2 μg GA/g. The total antioxidant capacity and total phenolic content obtained by in vitro measurements, using FRAP and Folin–Ciocalteu method, respectively, are shown in Table 2.

### 3.2. Baseline Characteristics

Fourteen participants completed the study, while two volunteers were unable to attend all study visit appointments. Subject characteristics at screening are presented in Table 3. Analysis of the food frequency questionnaires showed that the majority of subjects were consuming fruits 3–4 times per week, vegetables and tea 1–2 times per week, starch-rich foods every day, while they declared that they do not include herbal infusions in their diet (data not shown). Five participants were occasional light smokers (three of them were occasionally smoking 1–3 cigarettes per day, while two were occasionally smoking 4–7 cigarettes per day).

### 3.3. Effects of Control and Functional Meal Consumption on Blood Biomarkers

Table 4 shows the effect of acute consumption of the high-fat meal with spread cheese enriched with mountain tea and orange peel extract (Functional meal) or the same high-fat meal with the non-enriched spread cheese (Control meal) on serum and plasma biochemical parameters. Specifically, mean values of biomolecules serum glucose, cholesterol, triglycerides, HDL and LDL cholesterol, uric acid and plasma antioxidant capacity and resistance to oxidation are presented at baseline, and their differences from baseline at 30 min, 1.5 and 3 h after the consumption of the meal. Statistically significant differences were observed from baseline of the total antioxidant capacity at 30 min, 1.5 h and 3 h between Control and Functional meals.

### 3.4. Effect of Functional Meal Consumption on Plasma Total Antioxidant Capacity

Plasma total antioxidant capacity (TAC) differed significantly between the two groups at 3 h after meal consumption (*p* = 0.05), as presented in Table 5. Venous plasma TAC was significantly increased at 3 h following the meal with the Functional cheese compared with the Control meal, which significantly decreased (Δ 1.13 mmol/L and 0.56 mmol/L, respectively). A significant treatment x time interaction effect (*p* = 0.006) and a significant difference for change from baseline values (*p* = 0.024) was observed, as shown in Table 6. The significant changes in plasma TAC values in the different fractions, expressed as AUC (mmol/L%·h) after the test meals, are presented in 2a. There were no significant differences between TAC iAUCs following treatments (*p* = 0.447).

### 3.5. Effect of Functional Meal Consumption on Plasma Resistance to Oxidation

LTPOX differed significantly between the two groups at 30 min after meal consumption (*p* = 0.001). The resistance of plasma to copper-induced oxidation was significantly increased (*p* = 0.023) at 30 min following the meal with the Functional cheese compared with the Control meal, which was not significantly changed (*p* > 0.05). At the time points of 1.5 h and 3 h after meal consumption, a trend for higher values of LTPOX in the Functional group compared to the Control group was observed but was not statistically significant (*p* > 0.05).

### 3.6. Effect of Functional Meal Consumption on Serum Glucose, Lipids and Uric Acid Levels

**Figure 2:**
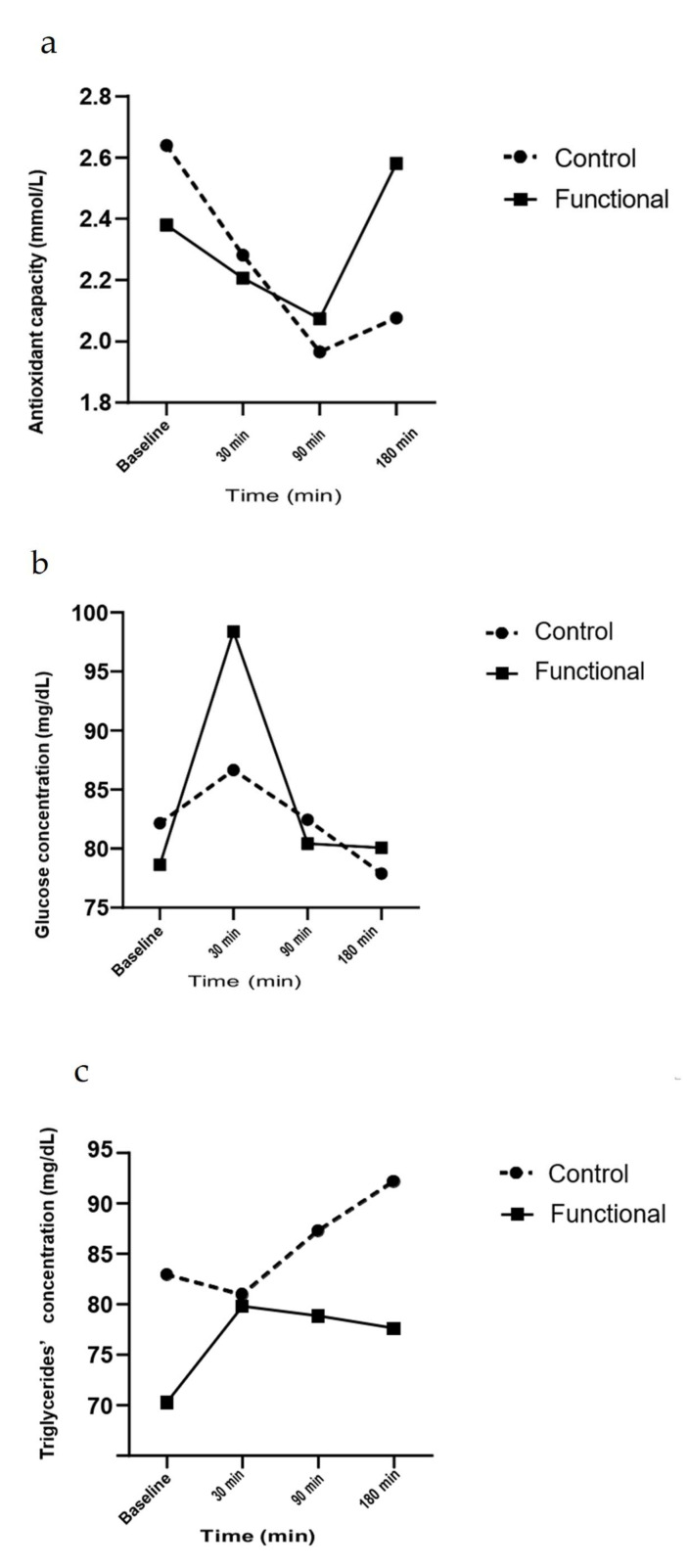
Incremental Area Under Curve (AUC) for antioxidant capacity, glucose and Triglycerides. (**a**) Venous plasma antioxidant capacity presented as Incremental Area Under Curve (AUC) following consumption of the Functional or the Control meal. (**b**) Serum glucose presented as AUC following consumption of the Functional or the Control meal. (**c**) Serum triglycerides presented as AUC, following consumption of the Functional or the Control meal. Values are presented as means ± SD, *n* = 14.

Serum glucose levels were significantly increased at 30 min after functional meal consumption (*p* = 0.024), while after Control meal consumption, were non significantly increased (Table 5). Serum glucose concentration differed for values’ changes 30 min from baseline (*p* = 0.05), with a significant treatment x time interaction effect (*p* = 0.034), as shown in Table 6. However, a tendency of reduction on the increase of glucose levels at 1.5 h after the Functional meal was observed (0.064), but the overall response to both meals did not differ from baseline values. The significant changes in serum glucose concentration in the different treatments, presented as AUC (mg/dL%·h) after both meals are given in Figure 2b.

Serum triglycerides concentration was significantly decreased at 1.5 h (Δ = −13.7) and 3 h (Δ = −12.5) after functional meal consumption (*p* = 0.001), as presented in Table 5. Serum triglycerides concentration differed significantly between treatments with a significant treatment interaction (*p* = 0.002), for values’ changes 30 min (*p* = 0.006), 1.5 h (*p* = 0.05) and 3 h (*p* = 0.04) from baseline (Table 6). Although, triglycerides levels 3 h after the Control meal are represented as non-significantly increased, while a tendency of reduction on triglycerides increase 3 h after functional meal consumption was observed (1.5 %, *p* = 0.062). The significant changes in serum triglycerides concentration in the different treatments, presented as AUC (mg/dL%·h), after both meals, are given in Figure 2c.

For the rest of the biomarkers tested (total, HDL, LDL cholesterol and uric acid) no statistically significant differences were observed for any interaction, as there was a similar response to the levels of these biomarkers after both meals’ consumption, as shown in Table 3, Table 6.

## 4. Discussion

The significance of the findings of the present study discussed herein is attributed to the scientific evidence that the postprandial rise in blood lipids and glucose and the subsequent induction of oxidative stress are associated with metabolic dysfunctions that possibly predict increased cardiovascular risk [3]. The main finding of the present study was the increase in plasma antioxidant capacity observed 3 h after the consumption of a meal rich in fat and carbohydrates, containing an enriched with mountain tea and orange peel extract spread cheese. On the contrary, no effect on plasma antioxidant capacity was observed after the ingestion of the same meal with the non-enriched cheese (Control). In addition, plasma residence to oxidation was increased at 30 min in the Functional meal, compared with the Control meal. The elevation in plasma antioxidant capacity observed after the ingestion of the meal with the novel cheese could be attributed to increased concentration of circulating polyphenolic metabolites, as it is speculated that they may be transferred to blood plasma [27,28]. Although several studies have investigated the antioxidant properties of mountain tea and orange peel extracts [17,29], there are no previous scientific data about the acute effect their consumption may pose on plasma oxidative status.

It has been demonstrated that mountain tea contains high levels of polyphenolic compounds such as phenylethanoid glycosides, flavonoids (mainly apigenin, luteolin, isoscutellarein and hypolaetin derivatives) and hydroxycinnamic acid derivatives, mainly ferulic acid and caffeic acid, which are reported to produce free radical scavenger actions [17]. Furthermore, it has been reported that the major phenolic compound identified by orange peel extract is hesperidin [30]. Our findings are in accordance with a recently published study by Anuyahong et al. [27], which demonstrated that the acute consumption of yogurt, enriched with anthocyanins from rice berry, increases plasma antioxidant capacity in comparison with a Control yogurt in healthy adults. This antioxidant activity was entirely associated with yogurt proteins and natural substances that provide bioactivity against free radical oxidation [27]. In addition, the results of our study corroborated the findings of Cardoso et al. 2015, who observed a significant increase in plasma antioxidant capacity of healthy subjects 1 h after the ingestion of a polyphenol-rich juçara juice [31]. Recent data from in vitro experiments also demonstrate an increase in the antioxidant activity of a polyphenol-enriched cheese in a simulated gastrointestinal environment [22]. Furthermore, scientific evidence demonstrates that elevated postprandial antioxidant activity may also be attributed to uric acid, an endogenous antioxidant which levels increase after the consumption of a meal and can contribute up to 60% of antioxidant activity [31,32]. In this study, however, we did not observe an increase in postprandial uric acid levels (*p* > 0.05).

The second important finding was the tendency of reduction on triglycerides’ levels increase 3 h after the intake of the Functional meal (*p* = 0.062). This could be attributed to better absorption of the fat provided by the meal, via inhibition of lipase activity, interfered by mountain tea and orange peel extract phenolic compounds [33]. Inhibiting effects on lipase activity of hesperidin, which represents the major phenolic compound identified in orange peel extract and also of hydroxycinnamic acid derivatives from mountain tea extract, have been reported previously [34]. The tendency of reduced increase of serum triglycerides concentrations following the meal with the novel cheese is in agreement with those reported by Hara Y et al., where postprandial serum triacyclglycerols elevation was significantly suppressed at 3 h and 5 h after the ingestion of a high-fat meal by drinking an oolong tea enriched beverage relative to the outcome when drinking the control beverage [35]. In addition, a previous study demonstrated that consumption of tea catechin supplements, as a flavonoid group, leads to a suppressing effect on the postprandial rise in plasma triacylglycerols in a dose-dependent manner, in comparison with the low-dose (control) [36]. The acute elevation of non-fasting triglycerides has been reported as a risk factor of cardiovascular disease, especially for arteriosclerosis. Therefore, the need for dietary interventions that suppress the postprandial rise in triglyceride levels is imperative [35]. The enrichment of a spread cheese with mountain tea and orange peel extract could contribute to this possible protective effect. However, further studies are required to consolidate this hypothesis, and additional measurements of circulating phenolic metabolites’ concentrations during the postprandial period need to be performed [37].

The third finding of this study was the tendency of reduction on the postprandial rise in glucose levels 1.5 h after the intake of the meal with the novel cheese relative to the consumption of the meal with the Control cheese (*p* = 0.064). This trend could be attributed to the fact that phytochemicals, provided by the infusion of mountain tea and orange peel extract, may inhibit carbohydrate digestion enzymes and may act against glycation and glycoxidation [16,27]. In vitro findings demonstrate that tea polyphenols inhibit intestinal glucose transport and also increase insulin secretion [38]. A similar effect on glucose levels’ attenuation after the consumption of meals rich in carbohydrates and polyphenols has been demonstrated in similar studies that investigate postprandial glycemia after consumption of different types of tea. As reported by Uchida et al., different varieties of black teas possess the ability to inhibit α-glucosidase activity, as total tea phenolic compounds were positively associated with inhibitory activity [39]. However, another study has shown that this inhibitory effect of black tea polyphenols is only observed in late-stage and not in early phase glycemia [40]. This finding could explain the observed in our study tendency for a lower rise in glucose levels without evidence of a significant decrease in glucose concentration, 3 h after the extract-enriched cheese intake, possibly attributed to the delay in the absorption of certain polyphenols.

The present study gives first-time, insight into the effect of the acute consumption of a spread cheese enriched with mountain tea and orange peel extract as part of a meal rich in fat and carbohydrates on the postprandial state in healthy subjects. Some limitations of the present study, though, need to be addressed. Plasma total antioxidant capacity is a common and useful tool to evaluate the oxidative stress status [41]. Nevertheless, other bioactive compounds in spread cheese (e.g., milk peptides) may influence postprandial total antioxidant capacity. Therefore, the quantification of the postprandial concentration of individual polyphenols after the consumption of the meal containing the novel spread cheese is important, as it may help evaluate the role of the enriched cheese in suppressing postprandial oxidative stress. Moreover, antioxidant action assessed with a single analysis cannot reflect the multiple reactions and mechanisms involved in oxidative stress. It only represents chemical reactivity under the specific conditions of analysis [28,42]. Furthermore, measurement of other biomarkers of oxidative stress, including ROS in leukocytes and platelets, markers based on ROS-induced modifications of lipids, DNA and proteins or enzymatic players of redox status may contribute to a more holistic evaluation of the oxidative status and the health-enhancing effects of the antioxidants [40]. Moreover, the adequacy of the sample size used in this study was statistically calculated, but sample size may have influenced the lack of statistical significance on the biomarkers tested, apart from antioxidant capacity. Studies with a higher number of participants should be performed to determine if a cheese enriched with mountain tea and orange peel extract can have more pronounced effects in the postprandial state. Furthermore, although moderate and heavy smokers were excluded, a major limitation of the study was the inclusion of five light, occasional smokers, given the fact that smoking may enhance oxidative stress not only through the production of reactive oxygen radicals in smoke but also through the weakening of the antioxidant defense systems [43]. Finally, a major limitation is that herein we studied the effect of acute consumption of enriched cheese in healthy volunteers as a postprandial preventive nutritional parameter; however, higher positive effects may be observed in subjects in cardiovascular risk, so future interventional studies in such patients could exact results of increased clinical interest.

In addition, we would like to underline that the development of novel functional foods is a multifactorial process in which, except for the possible exerted bioactivity and role on health promotion, several additional parameters should be considered. Organoleptic and sensorial characteristics, taste, smell, color, price and overall consumers acceptance are some of the important factors that determine the acceptance of food and its promotion on the market [44].

## 5. Conclusions

In conclusion, acute consumption of a spread cheese enriched with mountain tea–orange peel extract affected favorably postprandial biomolecules and metabolic biomarkers in healthy volunteers. Antioxidant activity was significantly increased 3 h after the consumption of the enriched cheese with a meal rich in fat and carbohydrates, compared to the consumption of the meal with the non-enriched cheese. A tendency to decrease the postprandial rise in glucose and triglyceride levels was also observed at 1.5 h and 3 h, respectively, after the consumption of the meal with the enriched cheese. This study designates a mountain tea–orange peel extract enriched cheese as a promising candidate for acute protective effects during the postprandial phase and may affect several biomolecules. These results warrant further studies involving consumption in a dose–response scheme or over a longer period of time, in both healthy and at risk for chronic disease populations, to verify its possible role as a functional food.

## Figures and Tables

**Table 1:** Dietary composition of the Control meal

**Nutrients of the Control Meal (130 g)**	
Energy (kcal)	445.89
Carbohydrates (g)	41.75
Fat, total (g)	27.45
Protein (g/kg)	8.41
Saturated fat (g)	16.06
Unsaturated fat (g)	9.65
Cholesterol (mg)	70.6
Dietary fiber, total (g)	1.92
Sugar, total (g)	4.5

**Table 2:** Total antioxidant capacity and total phenolic content obtained in vitro for sample extracts and spread cheeses.

Samples	Total Antioxidant Capacity (μmol FeSO_4_/mL)	Total Phenolic Content (μg GA/mL)
Mountain tea extract	11.54 ± 3.47	58.7 ± 1.7
Orange peel extract	6.7 ± 2.5	45.82 ± 9.1
Mountain tea–orange peel extract (massive extraction)	34.67 ± 6.5	49.9 ± 2.9
Control spread cheese	0.41 ± 0.14	3.65 ± 1.8
Spread cheese, enhanced with mountain tea–orange peel extract (massive extraction)	1.22 ± 0.22	6.2 ± 4.3

**Table 3:** Participants’ characteristics at baseline.

Participants’ Characteristics	
Participants (number)	14
Men (number)	6
Women (number)	8
Dietary supplementation (number of participants)	1
Physical activity medium or high (number of participants)	11
Age (years)	22.8 ± 1.9
Weight (kg)	65.6 ± 9.6
Height (cm)	169 ± 13.4
BMI	23 ± 0.6

Values for age, weight, height and BMI represents mean ± SD.

**Table 4:** Effects of Control and Functional meals on serum cholesterol, glucose, triglycerides, HDL and LDL cholesterol, uric acid levels, plasma antioxidant capacity and resistance to oxidation 30 min, 1.5 and 3 h after the consumption.

Serum Cholesterol (mg/dL)	Baseline	Δ 30 min	Δ 1.5 h	Δ 3 h
		(30 min–Baseline)	(1.5 h–Baseline)	(3 h–Baseline)
Control	127.8 ± 32.6	−15.05 ± 16.6	−7.37 ± 12.03	−4.1 ± 11.6
Functional	156 ± 0	6.3 ± 6.83	4.35 ± 16.8	2.6 ± 16
**Serum Glucose (mg/dL)**				
Control	84.2 ± 19.3	4.5 ± 24	7.25 ± 19.2	2.6 ± 8
Functional	78.2 ± 26.5	19.75 ± 13.8	4.64 ± 16	4.28 ± 8.2
**Serum Triglycerides (mg/dL)**				
Control	82.9 ± 32.9	−1.97 ± 22.7	11.6 ± 19.1	16.5 ± 24.3
Functional	70.3 ± 11.6	9.5 ± 7.45	13.7 ± 8.2	12.5 ± 6.05
**HDL cholesterol (mg/dL)**				
Control	56.6 ± 22	−4.15 ± 5.5	−1.8 ± 3.7	−1.18 ± 3.9
Functional	49.7 ± 31.2	2.87 ± 2.6	2.14 ± 4.1	2.85 ± 3.02
**LDL cholesterol (mg/dL)**				
Control	91.4 ± 38.9	−8.99 ± 8.6	−9.01 ± 9.5	−5.9 ± 10
Functional	82.5 ± 15,3	1.15 ± 4.7	−3.9 ± 13.2	−1.4 ± 11.9
**Uric Acid (mg/dL)**				
Control	5.5 ± 2	6.48 ± 17.8	1.08 ± 3.84	1 ± 2.3
Functional	7.2 ± 23.9	1.76 ± 2.4	0.74 ± 6.5	1.82 ± 8.5
**Lag Time of Plasma Oxidation (min)**				
Control	2.6 ± 0.95	−0.35 ± 1.08	−0.8 ± 1.02	−1.36 ± 1.18
Functional	2.4 ± 0.85	−0.17 ± 0.88	−0.44 ± 1.3	0.69 ± 1.25
**Plasma Antioxidant Capacity (mmoL FeSO_4_/L)**				
Control	94.5 ± 20.1 ^a^	−1.4 ± 4.5 ^a^	−3.4 ± 4.3 ^a^	−4.5 ± 6.7 ^a^
Functional	77.3 ± 20.5 ^a^	16.8 ± 9.5 ^b^	11.7 ± 12.6 ^b^	20.8 ± 23.7 ^b^

Δ indicates the difference of each timepoint from baseline. Values are means ± SD with 95% confidence intervals, *n* = 14. Δ Values in timepoints between Control and Functional with different letter represents statistically significant differences between the two interventions in the specific timepoint. Different letters (^a, b^) in a column indicate statistically significant differences between Control and Functional groups in the specific timepoint.

**Table 5:** Statistical significant effects of the Functional and Control meals on the biochemical parameters in specific timepoints.

Biomarker	*p* Value	Timepoint
**Plasma Total Antioxidant Capacity (mmol/L)**		
Control	0.015 *	3 h
Functional	0.05 *	
**Serum Glucose (mg/dL)**		
Control	0.124	30 min
Functional	0.024 *	
**Serum Triglycerides (mg/dL)**		
Control	0.082	1.5 h–3 h
Functional	0.001 *	

*p* values represents differences in the specific time point for each treatment (differences from baseline to time point); ANOVA Bonferoni test was performed. * Indicate statistical significant differences at *p* < or = 0.05.

**Table 6:** Differences between Control and Functional treatments over time and for changes from baseline, referring on the tested biomarkers.

**a. Differences over Time**						
**Biomarker**	**Treatment**	**Time**	Treatment * Time		**Paired-Samples *t*-Test**	
	*p* value ^a^	*p* value ^a^	*p* value ^a^		Timepoint	*p* value ^b^
Total antioxidant capacity (mmol/L)	0.471	0.382	0.007 *		3 h	0.005 *
Glucose (mg/dL)	0.131	0.064	0.145			*p* > 0.05
Triglycerides (mg/dL)	0.212	0.062	0.067			
Cholesterol (mg/dL)	0.313	0.936	0.37			
HDL cholesterol	0.766	0.172	0.247			
LDL cholesterol (mg/dL)	0.335	0.36	0.782			
Uric acid (mg/dL)	0.537	0.183	0.617			
**b. Differences from Baseline**						
**Biomarkers**	**Treatment**	**Time Period**	Treatment * Time Period	**Mean Difference**	**Paired-Samples *t*-Test**	
	*p* value ^c^	*p* value ^c^	*p* value ^c^		Time period	*p* value ^d^
Total Antioxidant Capacity (mmol/L)	0.19	0.275	0.006 *		Δ 3 h–Baseline	0.024 *
Glucose (mg/dL)	0.212	0.155	0.034 *	−9.5	Δ 30 min-Baseline	0.05 *
Triglycerides (mg/dL)	0.002 *	0.552	0.854		Δ 30 min-Baseline	0.006 *
					Δ 1.5 h–Baseline	0.05 *
Biomarkers					Δ 3 h–Baseline	0.04 *
Cholesterol (mg/dL)	0.232	0.728	0.788			*p* > 0.05
HDL cholesterol	0.13	0.066	0.385			
LDL cholesterol (mg/dL)	0.473	0.493	0.796			
Uric Acid (mg/dL)	0.145	0.188	0.588			

^a^*p* values represents differences in treatment effects, time effects and treatment x time interactions obtained by repeated-measures ANOVA of the changes between timepoints. * Indicate statistical significant differences at *p* < or = 0.05. ^b^ *p* value represents differences for treatment effects on timepoints obtained by paired-samples *t*-test of the changes between timepoints. ^c^ *p* values represents differences for treatment effects, time effects and treatment x time interactions, obtained by repeated-measures ANOVA of the changes up to 3 h from baseline (fasting, 0 h). * Indicate statistical significant differences at *p* < or = 0.05. ^d^ *p* value represents differences for treatment effects on time periods from baseline (Δ timepoint-baseline), obtained by paired-samples *t*-test of the changes up to 3 h from baseline (fasting, 0 h). * Indicate statistical significant differences at *p* < or = 0.05.

## Data Availability

The data presented in this study are available within this article.
